# The evolution and clinical importance of scar in hypertrophic cardiomyopathy - a 7 year CMR follow-up study

**DOI:** 10.1186/1532-429X-13-S1-P296

**Published:** 2011-02-02

**Authors:** Giovanni Quarta, Agata Grasso, Ferdinando Pasquale, Andrew S Flett, Dan Sado, Elena b, Cono Ariti, Sanjay K Prasad, Perry M Elliott, James C Moon

**Affiliations:** 1 University College London Hospitals Trust, London, UK; 2 Cardiovascular Magnetic Resonance, Royal Brompton and Harefield NHS Foundation Trust, London, UK; 3 Azienda Ospedaliera "Spedali Civili", Brescia, Italy, 4London School of Hygiene and Tropical Medicine, London, UK; 4 London School of Hygiene and Tropical Medicine, London, UK

## Introduction

Fibrosis is a key histopathological component of hypertrophic cardiomyopathy (HCM). Fibrosis, detected by CMR late gadolinium enhancement (LGE) contributes to diastolic dysfunction, ischaemia, atrial fibrillation, progression to heart failure, sudden cardiac death and is associated with exercise intolerance.

## Purpose

We sought to track long term changes in LGE by CMR in HCM and understand its clinical significance.

## Methods

Sixty patients with HCM underwent CMR in 2001-2003. Twelve of these patients (age 42.0 ± 18.7 years) underwent two CMRs at least 6 years apart (mean 7.4 ± 0.4 years). Follow up was not completed in the other 48 due to: 6 deaths, 1 cardiac transplantation, 17 device implantations (ICD, PPM), 8 declined; 6 lost to follow up; 4 had a normal follow-up scan; 6 index CMR not available. Morphological parameters (maximal wall thickness, LV mass, EF, LV volumes, LA size, SAM, LVOT obstruction) were measured by two independent observers. The entire short-axis LGE stack of images were analyzed quantitatively with MRI-MASS (Medis, Leiden, The Netherlands, FWHM technique to calculate LGE% total and per segment).

## Results

**Figure 1:**
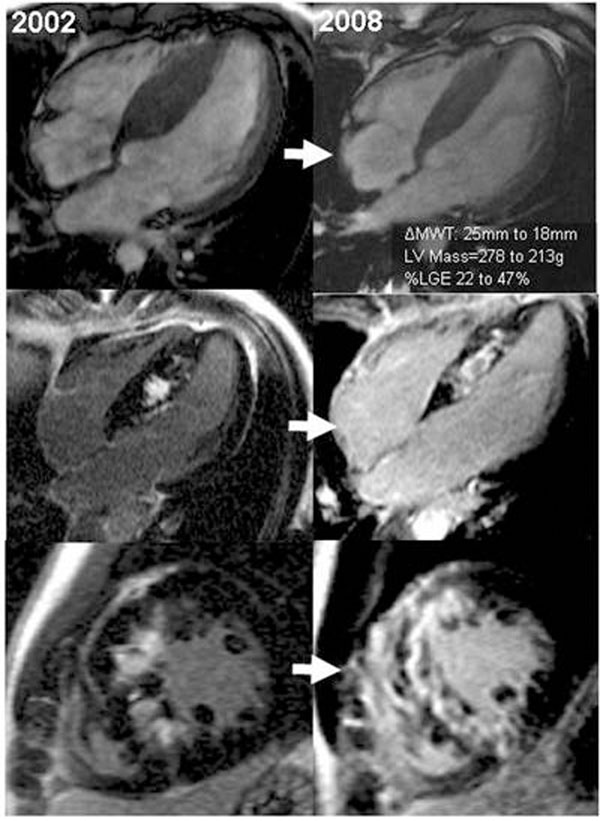


**Figure 2:**
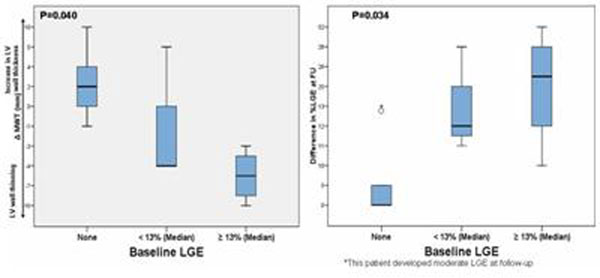


Five patients (42%) had no LGE at baseline and three of these had none at follow-up. In all other patients, LGE either appeared *de-novo* (two) or increased during follow-up (mean difference 20±14%, range 5-52%). Baseline LGE correlated with adverse remodelling: decrease in maximal wall thickness (linear association, p=0.001) and LV mass (linear association, p=0.05); LV EF tended to decrease and LVEDV tended to increase with increasing LGE at baseline, but did not reach statistical significance. The presence of LGE at baseline was strongly associated with worsening of LGE at follow up (R^2^=0.64, p=0.002). By segmental analysis, LGE increased on average by 13.8±22.5%, (p<0.001). The amount of LGE in the segment at baseline and the total percent of LGE at baseline were strongly associated with the amount of LGE at follow up (R^2^=0.54, p<0.001). Segmental baseline %LGE was strongly associated with the increase of LGE (p=0.016). Figures 1 and 2

## Conclusions

Over 7 years, HCM LGE increases. Baseline LGE predicts adverse remodelling. LGE begets LGE - with baseline LGE predicting future increases in LGE.

